# Assessing the Documentation of Social Determinants of Health for Lung Cancer Patients in Clinical Narratives

**DOI:** 10.3389/fpubh.2022.778463

**Published:** 2022-03-28

**Authors:** Zehao Yu, Xi Yang, Yi Guo, Jiang Bian, Yonghui Wu

**Affiliations:** Department of Health Outcomes and Biomedical Informatics, College of Medicine, University of Florida, Gainesville, FL, United States

**Keywords:** electronic health records, natural language processing, cancer, social determinants of health (SDOH), lung cancer

## Abstract

Social determinants of health (SDoH) are important factors associated with cancer risk and treatment outcomes. There is an increasing interest in exploring SDoH captured in electronic health records (EHRs) to assess cancer risk and outcomes; however, most SDoH are only captured in free-text clinical narratives such as physicians' notes that are not readily accessible. In this study, we applied a natural language processing (NLP) system to identify 15 categories of SDoH from a total of 10,855 lung cancer patients at the University of Florida Health. We aggregated the SDoH concepts into patient-level and assessed how each of the 15 categories of SDoH were documented in cancer patient's notes. To the best of our knowledge, this is one of the first studies to examine the documentation of SDoH in clinical narratives from a real-world lung cancer patient cohort. This study could guide future studies to better utilize SDoH information documented in clinical narratives.

## Introduction

As the second leading cause of death in the United States (US) (1), cancer has a long list of risk factors, ranging from biological traits to clinical characteristics to social determinants of health (SDoH) (2). In recent years, there is an increasing interest in examining how SDoH contribute to cancer risk and treatment outcomes (3). The Healthy People 2030 defined SDoH as “the conditions in the environments where people are born, live, learn, work, play, worship, and age that affect a wide range of health, functioning, and quality-of-life outcomes and risks” and categorized SDoH into 5 domains, including economic stability, education access and quality, healthcare access and quality, neighborhood and built environment, and social and community context (4). A recent study (5) reported that up to 75% of cancers occurrences are associated with SDoH rather than clinical factors. Other studies have shown that many SDoH contribute to individual cancer risk, influence the likelihood of survival, and affect cancer early prevention and health equity (2, 6, 7). A recent study (8) reported that SDoH factors such as poverty, lack of education, neighborhood disadvantage, and social isolation play important roles in breast cancer stage and survival. Many SDoH factors are also associated with the screening of cervical cancer, breast cancer, and lung cancer (9).

In the past decade, the rapid adoption of electronic health record (EHR) systems has made it possible to use the rich data elements (e.g., disease diagnoses, medications) captured in longitudinal patient's EHR data for cancer studies. However, it is challenging to extract SDoH from EHRs for assessing cancer outcomes as most SDoH were captured as free text in clinical notes rather than structured fields. In February 2018, the World Health Organization (WHO) defined structured codes to capture some of the SDoH. More specifically, the International Classification of Diseases, Tenth Revision, Clinical Modification (ICD-10-CM) Z codes (Z55–Z65) can be used to capture some SDoH. However, our previous study analyzed EHR data in a large clinical research network and showed that the use of SDoH Z codes is still quite low (10). Furthermore, it is unclear how well the SDoH information was documented in clinical notes for cancer patients. On the other hand, natural language processing (NLP) is the key technology to extract SDoH from clinical notes. NLP has been applied to extract various information such as diagnoses, lab tests, side effects from clinical narratives. We have explored many NLP models including state-of-the-art transformer-based NLP models in our previous studies (11–13). In a prior study (14), we have developed an NLP package to systematically extract SDoH from clinical notes using a subset of notes identified with a keyword matching pipeline.

In this study, we identified a cohort of lung cancer patients using ICD-9 and ICD-10 codes from the University of Florida Health (UF Health) system. We applied our NLP pipeline to systematically extract a total of 15 different categories of SDoH and examined the proportion of lung cancer patients who had various SDoH documented in clinical notes. This study is one of the earliest studies to examine how well the SDoH was documented in a real-world cohort of lung cancer patient, which will guide future studies exploring SDoH from clinical text for cancer studies.

## Methods

### Study Population: Lung Cancer Patients

In this study, we obtained clinical notes from the UF Health integrated data repository (IDR), a secure clinical data warehouse (CDW) that aggregates data from UF Health's various clinical and administrative information systems including the Epic electronic medical record (EMR) system. We used ICD-9 codes (162^*^) and ICD-10 codes (C34^*^) to identify a cohort of lung cancer patients from the UF Health IDR between 2011 and 2020. Patients who had at least one of the ICD-9 or ICD-10 codes and aged at least 18 years were included in the cohort. For each patient, we collected all types of clinical notes associated with the patient, which were used as the resource to extract SDoH concepts.

### An NLP Pipeline for Extracting SDoH

In our previous study (14), we created an SDoH corpus using 500 notes and developed an NLP pipeline that can extract 5 different categories of SDoH including gender, ethnicity, smoking, employment, and education from clinical narratives. In this study, we further extended the annotation with 10 new SDoH categories including race, alcohol use, drug use, marital status, occupation, language, physical activity, transportation, financial constraint, and social cohesion. Financial constraint indicates patients having a temporary or current financial problem but not in a poverty status (e.g., difficulty paying for the basics). Social cohesion indicates how well the patient connects to the society (e.g., attends religious service). Next, we re-trained the NLP model using this new corpus and developed an upgraded NLP pipeline that can extract a total number of 15 different categories of SDoH from clinical narratives. The transformer-based NLP model using the BERT architecture (15) was used in this study as it achieved the best performance in our previous study (14). BERT is a bidirectional transformer-based NLP model based on masked language modeling (MLM) and uses next-sentence prediction (NSP) to learn representations from text. This SDoH pipeline first identifies the SDoH concepts and then links them to various attributes including status, frequency, and negations. We reused the clinical transformer models developed in our previous study (11) implemented using the HuggingFace (16) package in PyTorch (17). We applied this NLP pipeline to all the clinical notes collected for our lung cancer patient cohort to extract SDoH concepts. Lastly, we aggregated the SDoH concepts at the patient level to assess the proportion of patients who had at least one SDoH concept documented in each of the 15 categories. When there were multiple SDoH instances extracted for one patients of the same category, we adopted majority voting strategy to keep the instance that most frequently documented in clinical notes.

## Results

We identified a total of 10,855 lung cancer patients in UF Health between 2011 and 2020 and collected a total of 1,798,409 clinical notes. Table 1 shows a summary of statistics for the demographics of this lung cancer cohort. Most patients (>95%) in this lung cancer cohort are >50 years old; there are more female patients than male and the majority race is White (>72%).

**Table 1:** Summary of statistics for the lung cancer cohort.

**Demographics**	**Sub-categories**	**Descriptive statistics (*N* = 10,855)**	**Percentage of the cohort (%)**
Age	18–30	79	0.73
	30–40	112	1.03
	40–50	283	2.61
	50–60	1,433	13.20
	60–70	3,441	31.70
	70–80	3,568	32.87
	>80	1,939	17.86
Gender	Female	4,643	57.23
	Male	6,212	42.77
Race	White	7,834	72.17
	Africa American	1,517	13.98
	Asian	88	0.81
	American Indian or Alaska Native or Native Hawaiian or Other Pacific Islander	18	0.17
	Multi-Race	19	0.18
	Other*	1,379	12.70
Ethnics	Hispanic or Latino	210	1.93
	Not Hispanic or Latino	9,459	87.14
	Other*	1,186	10.93

Based on our previous annotation of 5 SDoH categories using 500 clinical notes (14), we further annotated additional 10 SDoH categories and extended the previous annotation from 1,876 SDoH concepts of 5 categories to a total of 5,015 concepts of 15 different SDoH categories. Following the standard NLP development procedure, we divided the annotation into a training set and a test set using a ratio of 4:1. We used the training set to optimize the parameter of a BERT model and used the test set to calculate evaluation scores. We reused the same experiment settings for batch size and learning rate identified from our previous study (14). Using the new extended corpus, the performance (micro average F1 score for all SDoH categories) of our SDoH NLP pipeline based on the BERT model improved from 0.8791 (precision: 0.8848 and recall: 0.8734) to 0.9216 (precision: 0.9298, recall: 0.9136).

We applied the BERT-based NLP pipeline and identified a total number of 5,408,148 SDoH concepts from 1,798,409 clinical notes of 10,855 lung cancer patients. We then aggregated the SDoH concepts at the patient level and calculated the distribution of SDoH concepts for each category. Majority voting was used when there were multiple SDoH instances identified for one SDoH category. Table 2 shows the total number of SDoH concepts identified in each SDoH category and the percentage of patients with at least one SDoH concept in each category. Among the 15 SDoH categories, 3 categories (i.e., gender, alcohol use, and drug use) were extremely frequent-documented in the lung cancer patients, where over 90% of the patients in this cohort had at least one SDoH documented; 5 categories (i.e., marital status, education, occupation, smoking, race) were frequent-documented, where over 70% of the patients had at least one SDoH documented; 7 categories (i.e., ethnicity, language, physical activity, transportation, financial constraint, social cohesion, employment status) were not frequent-documented, where <60% of the patients had at least one SDoH documented.

**Table 2:** Social determinants of health (SDoH) concepts identified from the lung cancer patient cohort.

**SDoH category**	**Total number of concepts detected by NLP**	**Total number of patients has at least one SDoH**	**Percentage of patients has at least one SDoH for current category (%)**
Gender	843,066	9,552	98.7
Alcohol use	223,214	9,195	95.0
Drug use	180,309	8,756	90.5
Marital status	167,457	8,655	89.5
Education	167,018	8,463	87.5
Occupation	142,306	8,345	86.3
Smoking	132,833	7,639	79.0
Race	144,980	7,376	76.2
Ethnicity	86,789	5,231	54.1
Language	83,539	5,173	53.5
Physical activity	55,842	3,092	32.0
Transportation	24,191	2,877	29.7
Financial constraint	113,220	2,766	28.6
Social cohesion	9,170	2,727	28.2
Employment status	843,066	2,110	21.8

## Discussion

Many SDoH are associated with cancer risk and cancer treatment outcomes. Yet, information related to SDoH is often unavailable in structured EHRs but is often documented in clinical notes as free text, making it challenging to examine SDoH in cancer research. In this study, we identified a cohort of lung cancer patients and applied our NLP system to extract SDoH concepts from 15 categories of SDoH. We examined the distribution of SDoH in each category and evaluated how frequent SDoH was documented for categories. This study will guide future cancer studies that aim to explore SDoH information from clinical notes.

## Data Availability Statement

The data analyzed in this study is subject to the following licenses/restrictions: we obtained clinical notes from the UF Health integrated data repository (IDR), a secure clinical data warehouse (CDW) that aggregates data from UF Health's various clinical and administrative information systems including the Epic electronic medical record (EMR) system. Requests to access these datasets should be directed to UF CSTI, info@ctsi.ufl.edu.

## Author Contributions

JB, YG, and YW were responsible for the overall design, development, and evaluation of this study. XY collected the data used in this study and involved in the results analysis. ZY conducted the experiments and data analysis. YW did the initial drafts and revisions of the manuscript. All authors reviewed the manuscript critically for scientific content and gave final approval of the manuscript for publication.

## Funding

This study was partially supported by a Patient-Centered Outcomes Research Institute® (PCORI®) Award (ME-2018C3-14754), a Grant from National Institute on Aging 1R56AG 069880, a Grant from the National Cancer Institute, 1R01CA246418 R01, and the Cancer Informatics and eHealth core jointly supported by the UF Health Cancer Center and the UF Clinical and Translational Science Institute.

## Author Disclaimer

The content is solely the responsibility of the authors and does not necessarily represent the official views of the funding institutions.

## Conflict of Interest

The authors declare that the research was conducted in the absence of any commercial or financial relationships that could be construed as a potential conflict of interest.

## Publisher's Note

All claims expressed in this article are solely those of the authors and do not necessarily represent those of their affiliated organizations, or those of the publisher, the editors and the reviewers. Any product that may be evaluated in this article, or claim that may be made by its manufacturer, is not guaranteed or endorsed by the publisher.
